# Effects of waterborne nickel on the physiological and immunological parameters of the Pacific abalone *Haliotis discus hannai* during thermal stress

**DOI:** 10.1007/s11356-015-4597-1

**Published:** 2015-05-06

**Authors:** Eun Young Min, Yong-Joo Cha, Ju-Chan Kang

**Affiliations:** Institute of Fisheries Science, Pukyong National University, Busan, 619-911 Korea; Departments of Aquatic Life Medicine, Pukyong National University, Busan, 608-737 Korea

**Keywords:** Nickel, Thermal stress, LC_50_, Hemolymph, Immunology, Hematology, THC, Lysozyme, PO, Phagocytosis, *Haliotis discus hannai*

## Abstract

In this study, the 96-h LC_50_ at 22 and 26 °C values was 28.591 and 11.761 mg/L, respectively, for NiCl_2_ exposure in the abalone. The alteration of physiological and immune–toxicological parameters such as the total hemocyte count (THC), lysozyme, phenoloxidase (PO), and phagocytosis activity was measured in the abalone exposed to nickel (200 and 400 μg/L) under thermal stress for 96 h. In this study, Mg and THC decreased, while Ca, lysozyme, PO, and phagocytosis activity increased in the hemolymph of Pacific abalone exposed to NiCl_2_ when compared to a control at both 22 and 26 °C. However, these parameters were not affected by a rise in temperature from 22 to 26 °C in non-exposed groups. Our results showed that NiCl_2_ below 400 μg/L was able to stimulate immune responses in abalone. However, complex stressors, thermal changes, or NiCl_2_ can modify the immunological response and lead to changes in the physiology of host–pollutant interactions in the abalone.

## Introduction

The average sea surface temperature has increased in the last 100 years, and these changes are ongoing (Hoegh-Guldberg and Bruno 2010). Recently, climate change has been implicated in the increasing frequency and severity of disease outbreaks in marine environments (Harvell et al. 2008; Lejeusne et al. 2010). For example, from July to early September 2012, mass mortality occurred in several fish species, particularly the black rockfish *Sebastes schlegeli* raised in floating fish cages along the coast of Gyeongsangnam-do, Korea. A rapid rise in water temperature was confirmed to be the cause of damage to 1,802,000 fishes (Lee et al. 2013). However, the cause of this abnormal mortality being just the high temperature in summer, with no obvious indication of disease, is doubtful.

Temperature is one of the main environmental factors that can cause significant changes in the physiology of ectothermic organisms and thus affects their sensitivity to xenobiotic substances. Some metals are hazardous to aquatic organisms due to their long-term persistence, severe toxicity, and bioaccumulation properties (Atchison et al. 1987). Heavy metal contaminants influence the increased incidence of disease by adversely affecting immunity, thereby enhancing susceptibility to stress and infection (Auffret et al. 2002), because heavy metals are themselves immune–toxic substances (Gagne et al. 2008; Vijayavel et al. 2009). However, factors such as temperature and xenobiotic substances do not act as the sole stressor alone and may act in combination to alter normal immune function, resulting in adverse health outcomes in aquatic organisms (Wanger et al. 1997; Ortuno et al. 2002; Prophete et al. 2006). Accordingly, further research is needed to assess which factors in hot summers are responsible for the increased mortality in heavy-metal-polluted aquatic farms.

Nickel (Ni) is an important contaminant present at elevated concentrations in aquatic ecosystem that is currently impacted by the many industrial uses and natural ways (Eisler 1998; Muyssen et al. 2004). Ni concentrations, which are typically below 10 μg/L in unimpacted water, may reach as high as several hundreds to 1000 μg/L in highly contaminated water (Eisler 1998). Although Ni is considered to be an essential for a wide variety of animals species, its essentiality to aquatic animals is not fully established (Muyssen et al. 2004). Several studies reported a Ni-related depression of immune system both in vertebrates and invertebrates (Eisler 1998; Harkin et al. 2003; Vijayavel et al. 2009; Sun et al. 2011). For example, the exposure of the mud crab *Scylla serrata* to Ni has been reported to modulate the hemocytic defense system (Vijayavel et al. 2009). Also, the fish immune responses seem to be a sensitive target for the suppressive effects of Ni, decreasing the number of lymphocytes (Zelikoff 1994; Zelikoff et al. 1996). In addition, Ni has been well studied in mammals due to its toxic effects on the immune system (Zhang et al. 2008).

A marine gastropod, the Pacific abalone *Haliotis discus hannai*, is an important fishery and food resource farmed in the Americas, Africa, Asia, and Australia (Nguyen et al. 2013). Previous studies have shown that physical stresses such as alterations in temperature, salinity, and oxygen appear to exert a great impact upon immune defense responses in several abalone species (Martello et al. 2000; Malham et al. 2003; Cheng et al. 2004a, b, c, d, e; Zoysa et al. 2009). The immunological biomarkers, effects, or susceptibility of exposure are complementary, and understanding the overall health impact of toxicants is important. Furthermore, gastropods and bivalve mollusks can be used as indicators of marine metallic pollution because they accumulate metals in their tissues in proportion to the degree of environmental contamination (Elder and Mattraw 1984).

In the aquatic environment, organisms, especially in the case of abalones farmed in cages that cannot move away from a detected danger, simultaneously undergo various physical and chemical stimulations. Therefore, the aims of this study were to study the combined effects of water temperature and a metal (Ni) on acute toxicity and survival and to consider the sublethal effects of Ni on immune–toxicological biomarkers in *H. discus hannai*.

## Materials and methods

### Temperature acclimations

Pacific abalone (*H. discus hannai*; body mass 23.147 ± 0.83 g, shell length 6.041 ± 0.07 cm) were obtained from a commercial farm (Namhae, Korea). Abalone specimens were held for 2 weeks in seawater at 22 °C to ensure that all individuals were healthy and feeding and also to reset the thermal history of the animals prior to initiating temperature acclimations. The animals were fed on a marine macroalgae diet of *Laminaria digitata* twice daily. The water temperature was adjusted from ambient at a rate of ±1 °C/day until a final temperature of 26 °C was reached. The acclimation period commenced once the final temperature had been sustained for 24 h and animals were feeding, while showing no sign of stress. Animals were acclimated to 22 or 26 °C under laboratory conditions during 96 h before the experiment (Table 1).

**Table 1:** 20 % and 50 % lethal concentration (LC_20_ and LC_50_ with 95 % upper and lower confidence limits) of *H. discus hannai* Ino in different NiCl_2_ concentrations at 22 and 26 °C for 96 h calculated by probit analysis

Water temperatures (°C)	Probit analysis	Estimated values (mg/L)	95 % confidence limit	
			Upper limit	Lower limit
22	LC_20_	9.929	−21.196	22.637
	LC_50_	28.591	14.747	49.526
26	LC_20_	4.116	−5.428	8.169
	LC_50_	11.761	7.588	16.764

Control and NiCl_2_ concentration lower than 5 mg/L did not have any mortality until the end of the exposure periods

### Acute toxicity study

This test was conducted in accordance with standardized methods (ASTM 1980). A 96-h LC_50_ (median lethal concentration) was measured for abalone at our test water temperatures: 22 and 26 °C using the static renewal method. On a daily basis, a 100 % of the water change was performed with test solutions that were made 24 h prior to use to allow for metal equilibration. At time 0, the exposure tanks were spiked with a concentrated stock prepared from Ni(II) chloride hexahydrate (NiCl_2,_ purity 97 %; Sigma-Aldrich, St. Louis, MO, USA) dissolved in double-distilled water. Abalone (*n* = 10 per tank) were transferred to one of eight 30-L tanks (including one control and seven different NiCl_2_ concentrations, nominally 0.5, 1, 5, 10, 20, 40, and 80 mg/L), each containing 20 L of well-aerated seawater under laboratory conditions. The water quality parameters measured for the bioassay were as follows: pH, 8.10 ± 0.2; salinity, 33.50 ± 0.6‰; and dissolved oxygen (DO), 7.14 ± 0.3 mg/L. All experiments were conducted at a room temperature of 20 ± 0.5 °C under a 12-h light/12-h dark cycle. No feed was provided during the 96-h test period. Dead animals were removed immediately from the test tank. Three replicates were performed for each concentration. The percentage mortality of animals was noted after 96 h, and the 96-h LC_50_ value was recorded and tested using a probit analysis program as described by Finney (1971).

### Sublethal toxicity study

To assess the changes in biomarkers, *H. discus hannai* were divided into nine groups of five specimens each. Group 1–2 animals were reared individually in normal seawater at 22 and 26 °C. Group 3–4 and 5–6 animals were exposed to seawater containing 100 and 400 μg/L NiCl_2_ at 22 and 26 °C, respectively. Experimental concentrations were sublethal at which 0 % mortality occurred by 96 h. Glass aquaria (28 cm × 50 cm × 30 cm) were used in the experiments. The test solution and seawater were renewed daily to provide a constant effect of Ni on the animals. The animals were fed on a marine macroalgae diet of *L. digitata* during the 96-h experimental period. After 96 h, the experiment was terminated and the animals were killed to assess the biochemical and immunotoxic parameters.

### Analysis of hematological and immunological parameters

#### Hemolymph collection

Hemolymph was withdrawn from the cephalic arterial sinus located at the anterior part of the muscle using a 26-gauge needle attached to a sterile plastic syringe containing ice-cold Tris-buffered saline (TBS; 50 mM Tris, 370 mM NaCl; pH 8.4), which prevents the clumping of hemocytes. Hemolymph from each animal was transferred into a vial and kept on ice. Approximately 200 μL of hemolymph samples was collected separately in 500 μL TBS and centrifuged at 200×*g* for 10 min at 4 °C. The supernatant plasma was aliquoted separately and used for phenoloxidase (PO) and biochemical assays. The resulting hemocyte pellet was resuspended in an equal volume of TBS, and the hemocytes were used for the phagocytosis assay.

#### Hemolymph biochemical parameters

Plasma samples were analyzed for inorganic substances, organic substances, and enzyme activity using a clinical kit (Asan Pharmaceutical Co., Ltd., Seoul, Korea). In the inorganic substance assay, calcium (Ca) and magnesium (Mg) were analyzed using the *o*-cresolphthalein complexone and xylidyl blue methods. In the organic substance assay, glucose and total proteins were analyzed using the glucose oxidase/peroxidase (GOD–POD) and biuret methods. In the enzyme activity assay, alkaline phosphatase (ALP) was analyzed using the Kind and King technique.

### Total hemocyte count

An aliquot (200 μL) of hemolymph was collected in a prechilled vial containing 0.2 mL of sodium cacodylate-based anticoagulant (4.28 g of sodium cacodylate added to 90 mL of distilled water, pH 7.0; 400 μL of stock 25 % glutaraldehyde solution added and volume adjusted to 100 mL with distilled water) preloaded in a 1-mL syringe to count the total hemocytes using a hemocytometer (Neubauer, improved; Superior Ltd., Lauda-Königshofen, Germany) mounted in a microscope (CX40; Olympus, Shinjuku, Japan).

### Lysozyme activity

The lysozyme concentration was calculated by measuring enzyme activity. Lysozyme activity was determined by a turbidimetric method (Ellis 1990) using *Micrococcus lysodeikticus* (Sigma-Aldrich) as a substrate (0.2 mg/mL 0.05 M phosphate buffer; pH 6.6 for kidney samples and pH 7.4 for plasma). A standard curve was made with a lyophilized hen egg white lysozyme (Sigma-Aldrich), and the rate of change in turbidity was measured at 0.5- and 4.5-min intervals at 530 nm. The result was expressed as microgram per milliliter and microgram per gram equivalent of hen egg white lysozyme activity.

### Phenoloxidase activity

PO activity was measured according to the method described by Asokan et al. (1997). Briefly, 100 μL of 2 mM l-DOPA was added to 200 μL of plasma in a 96-well flat-bottomed plate, and the optical density was measured at 490 nm for 10 min in a microplate reader (Zenyth 200rt; Anthos Labtec Instruments GmbH, Salzburg, Austria). One unit was defined as an absorbance change of 0.001 min/mg protein (U/mg protein/min).

### In vitro phagocytosis

Phagocytosis was measured using a cytoselect 96-well Phagocytosis Assay kit (Cell Biolabs, Inc, San Diego, CA, USA) according to the manufacturer’s instructions. One hundred microliters of plasma was placed in a 96-well plate, and each reagent was added sequentially. The optical density was measured at 450 nm in a Zenyth 200rt Microplate Reader.

### Statistical analysis

Three experimental chambers were set up, each containing ten animals. Statistical analyses were performed using the SPSS/PC+ statistical package (SPSS Inc, Chicago, IL, USA). Significant differences between groups were identified using one-way analysis of variance (ANOVA) and Duncan’s test for multiple comparisons. The significance level was set at *P* < 0.05. Water temperature and physiological responses were examined with the two-way ANOVA followed by Tukey’s HSD post hoc tests after testing for normality and homogeneity of the data. The correlation between physiological responses was assessed with the Spearman correlation coefficient.

## Results and discussion

Although various studies have established relationships between water temperature and outbreaks of infectious diseases in abalone (Lee et al. 2001; Braid et al. 2005; Dang et al. 2012), the effects of a combination of stressors in the marine environment remain elusive. The impact of the increased frequency of extreme thermal events on the physiological and immunological responses against the toxic effects of heavy metals remains unknown in abalone. Therefore, the aim of this study was to examine the effect of two commonly occurring aquatic stressors on the immune response of a commercially important Pacific abalone.

In this study, NiCl_2_ caused acute toxicity in a concentration-dependent manner in *H. discus hannai*. Using the data generated from the concentration–response experiments, the 96-h LC_20_ and LC_50_ values and their 95 % confidence limit levels were calculated (Table 1). The experimental conditions produced no mortality in the control at 22 and 26 °C. When administered at 40 and 80 mg/L at both 22 and 26 °C, NiCl_2_ induced 100 % cumulative mortality in the three replicate groups within 4 days of exposure. The 96-h LC_50_ at 22 and 26 °C for the abalone was 28.591 and 11.761 mg/L, respectively.

Previous studies have reported the LC_50_ of Ni in various fisheries (Saxena and Parashari 1983; Alam and Maughan 1982; Virk and Sharma 1995; Buhl and Hamilton 1991; Khangarot and Ray 1990), but little information exists in the literature regarding the toxic effects of Ni on invertebrates. Reported values of the 96-h LC_50_ for NiCl_2_ exposure are 2.26 mg/L in the mud crab *S. serrata*, 8.46 mg/L in the freshwater snail *Melanoides tuberculata*, and 112 mg/L in the pink shrimp *Penaeus duorarum* (Othman et al. 2012; Vijayavel et al. 2009). The variation in the LC_50_ values in aquatic organisms reported in these studies might be attributable to the species; size; age; and water quality parameters such as temperature, hardness, pH, and oxygen level (Rand and Petrocelli 1985). The results of this study, as shown in Table 1, indicate that temperature had a considerable effect on the survival of abalone exposed to NiCl_2_. Temperature is the most important natural factor affecting the toxicity of pollutants, partly because of its direct and immediate effects on metabolic processes (Sjursen and Holmstrup 2004; Khan et al. 2007). Unfavorable temperatures may affect uptake, elimination, and detoxification rates through an influence on the metabolic, locomotor, and feeding activities of organisms (Donker et al. 1998).

The mode of toxicity of most waterborne metals involves the disruption of the immune defense system (Pipe et al. 1999), but the mechanism of Ni toxicity is more unclear, particularly in marine environments. For example, a study of cadmium (Cd) showed no significant immunotoxic effects on the hemocytes of the Pacific oyster *Crassostrea gigas*, while mercury (Hg) inhibited PO activity and generated high mortality in these cells in vitro (Gagnaire et al. 2004). In the clam *Mya arenaria*, the hemocyte phagocytic activity significantly decreased due to the high levels of zinc (Zn), Cd, Hg, mercury chloride (HgCl), and nitrate (NO_3_^−^; Brousseau et al. 2000).

In mollusks, the immune defense system mainly depends on innate immunity and more specifically on hemocytes circulating in the hemolymph, which are also thought to be important antimicrobial effector cells. Following phagocytosis, one of the important roles of hemolymph in the invertebrate defense system is as an early internal defense mechanism against invaders by circulating hemocytes. Any decrease in the total hemocyte count (THC) and phagocytic activity due to xenobiotic chemicals could lead to a decrease in the defense response against pathogens (Yue et al. 2010). Hemocytes also have important roles in lysosomal enzyme activity, anti-inflammation, wound repair, and the production of reactive oxygen species (ROS).

In this study, two concentrations (100 and 400 μg/L) of NiCl_2_ were used to assess the biochemical and immunological parameters in *H. discus hannai*. The parameters were modulated by NiCl_2_ exposure or change of water temperature (Table 2). NiCl_2_ exposure, change of water temperature, and their interaction affected total protein levels and the lysozyme activities in *H. discus hannai* (Table 2). The effect of NiCl_2_ on Ca, Mg, total protein, glucose, and ALP-S in the hemolymph of abalone is shown in Table 2. The level of Ca increased significantly with a decrease in the level of Mg in the hemolymph of *H. discus hannai*, depending on the water temperature and NiCl_2_ concentration compared to the control at 22 and 26 °C (*P* < 0.05).

**Table 2:** Biochemical analysis of hemolymph in *H. discus hannai* Ino in different NiCl_2_ concentrations at 22 and 26 °C for 96 h

	Water temperatures (°C)	Exposure concentration (μg/L)		
		Control	100	400
Ca (mg/dL)	22	4.270 ± 0.389^a^	5.506 ± 0.065^ab^	6.180 ± 0.324^ab^
	26	6.854 ± 0.065^b^	9.438 ± 1.557^c^	9.438 ± 0.778^c^
Mg (mg/dL)	22	13.102 ± 0.386^c^	12.834 ± 0.618^c^	13.235 ± 0.463^c^
	26	11.832 ± 0.039^bc^	10.829 ± 0.618^ab^	10.361 ± 0.039^a^
Total protein (g/dL)	22	2.416 ± 0.005^a^	2.426 ± 0.001^a^	2.417 ± 0.005^a^
	26	2.508 ± 0.037^b^	2.453 ± 0.005^ab^	2.407 ± 0.001^a^
Glucose (mg/dL)	22	26.238 ± 0.286^a^	26.733 ± 0.001^ab^	27.228 ± 0.286^ab^
	26	27.723 ± 0.286^b^	27.475 ± 0.661^b^	27.227 ± 0.285^ab^
ALP-S (K-A)	22	6.316 ± 0.058^a^	6.391 ± 0.043^ab^	6.541 ± 0.043^b^
	26	6.391 ± 0.043^ab^	6.541 ± 0.043^b^	6.466 ± 0.058^ab^

Each value represents a mean value ± SD of three replicates (*n* = 10). Values with different superscripts are significantly different (*P* < 0.05) as determined by Duncan‘s multiple range test

The mode of toxicity of most waterborne metals involves the disruption of ion regulation (Bielmyer et al. 2013). The physiological mechanisms of Ni toxicity in aquatic organisms are yet to be fully understood (Niyogi et al. 2014). In aquatic invertebrates, Ni appears to act more like an ion regulatory toxicant, particularly in acute exposures. In *Daphnia magna*, Ni acts as the Mg^2+^ antagonist, thereby disrupting Mg^2+^ homeostasis and causing a decrease in Mg^2+^ levels following both acute and chronic exposure (Pane et al. 2003). Alternatively, in fish, Ni has been found to act primarily as a respiratory, rather than an ion regulatory, toxicant in both acute and chronic exposures (Pane et al. 2003, 2004). In this study, NiCl_2_ appeared to disrupt plasma Ca^2+^ and Mg^2+^ homeostasis because increased concentrations of NiCl_2_ induced physiological stress under unfavorable temperature. In addition, Ca^2+^ is known to play a role in apoptosis. Previous study has suggested that Ca^2+^ accumulation in the cytoplasm disrupts Ca^2+^ ion homeostasis in crab (*Scylla* sp.), subsequently causing the dysfunction of mitochondria and endoplasmic reticulum and finally leading to the apoptosis of muscle cells under thermal stress (Kong et al. 2012).

**Fig. 1:**
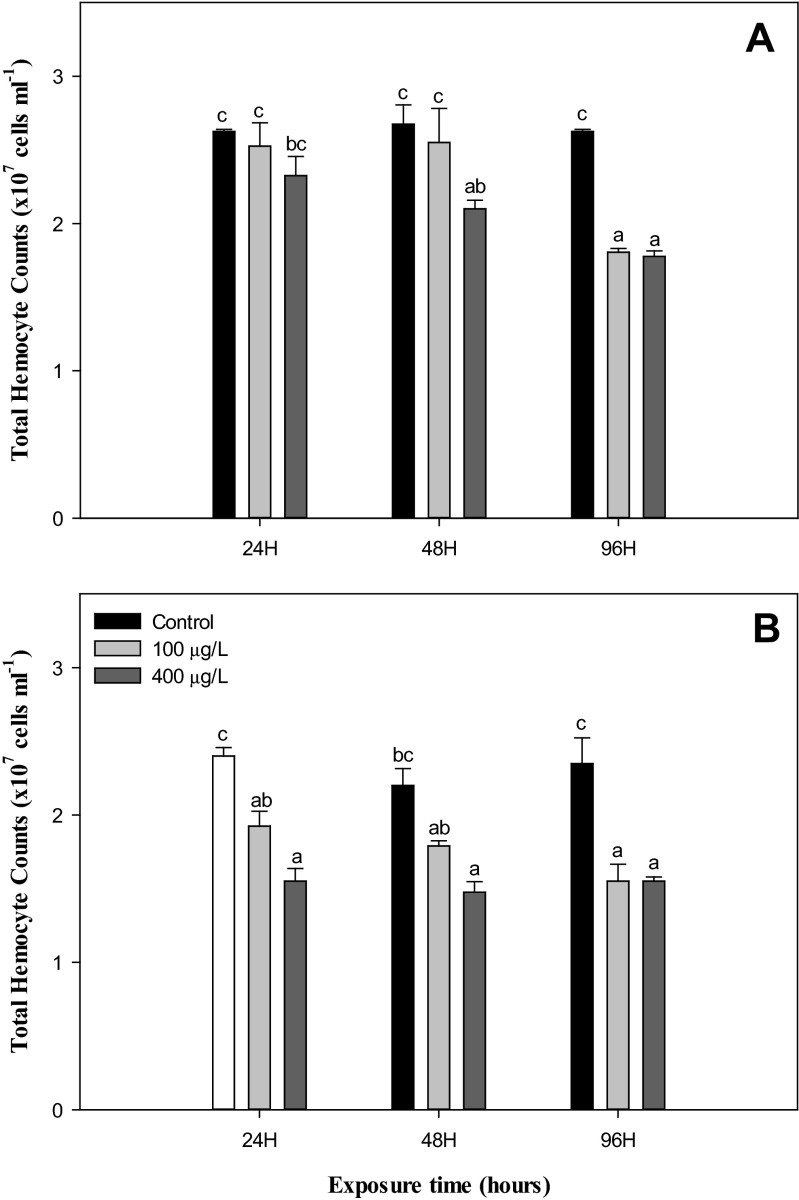
Total hemocyte counts in abalone, *H. discus hannai* Ino, exposed to various NiCl_2_ concentrations at 22 °C (**a**) and 26 °C (**b**) for 96 h. Each point represents a mean value ± SD of three replicates. *Vertical bar* denotes a standard error (*n* = 5). Values with *different superscripts* are significantly different (*P* < 0.05) as determined by Duncan’s multiple range test

**Fig. 2:**
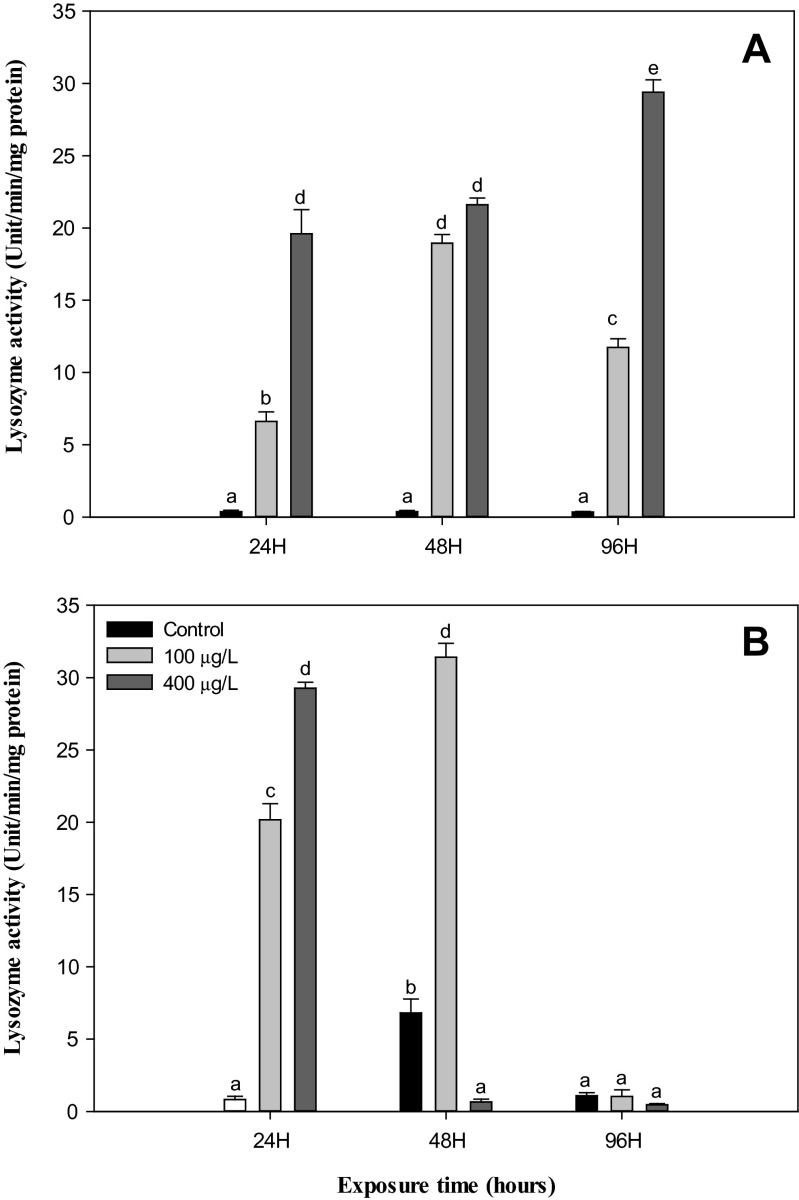
Lysozyme activities of hemocytes in abalone, *H. discus hannai* Ino, exposed to various NiCl_2_ concentrations at 22 °C (**a**) and 26 °C (**b**) for 96 h. Each point represents a mean value ± SD of three replicates. *Vertical bar* denotes a standard error (*n* = 5). Values with *different superscripts* are significantly different (*P* < 0.05) as determined by Duncan’s multiple range test

**Fig. 3:**
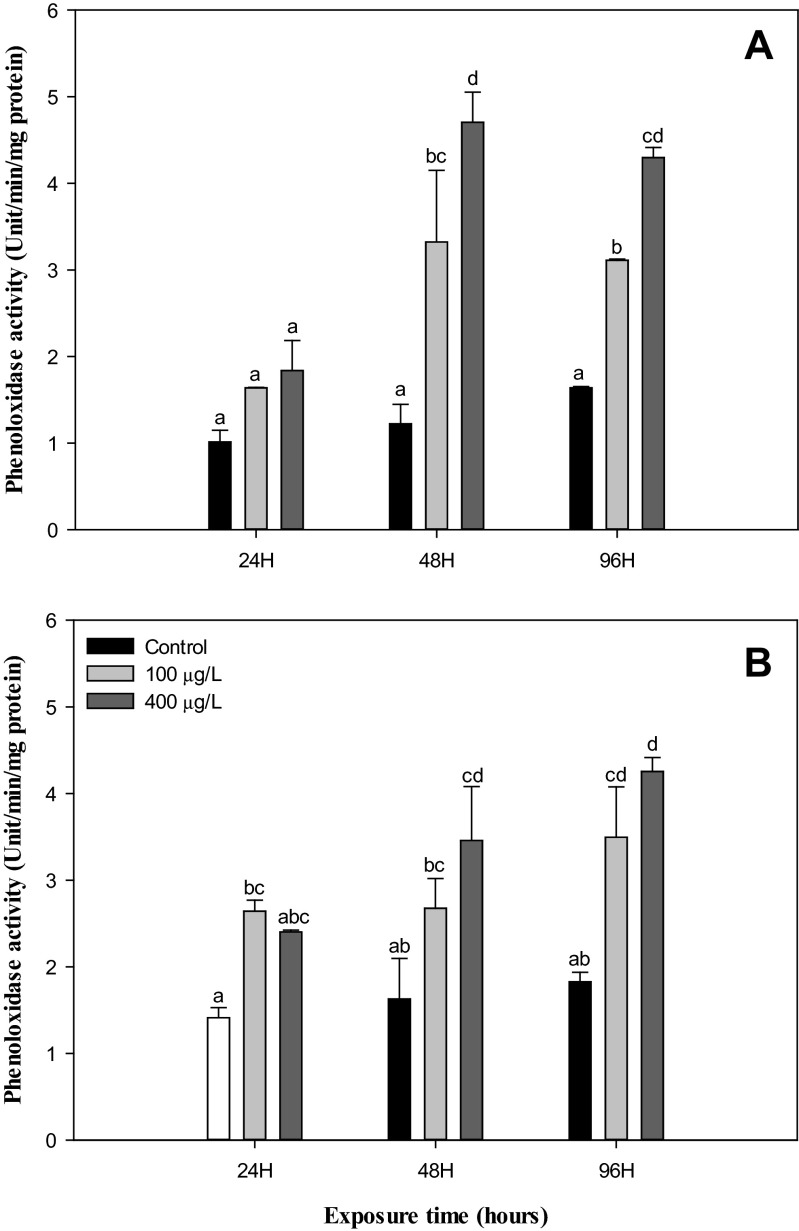
Phenoloxidase activity of hemocytes in abalone, *H. discus hannai* Ino, exposed to various NiCl_2_ concentrations at 22 °C (**a**) and 26 °C (**b**) for 96 h. Each point represents a mean value ± SD of three replicates. *Vertical bar* denotes a standard error (*n* = 10). Values with *different superscripts* are significantly different (*P* < 0.05) as determined by Duncan’s multiple range test

**Fig. 4:**
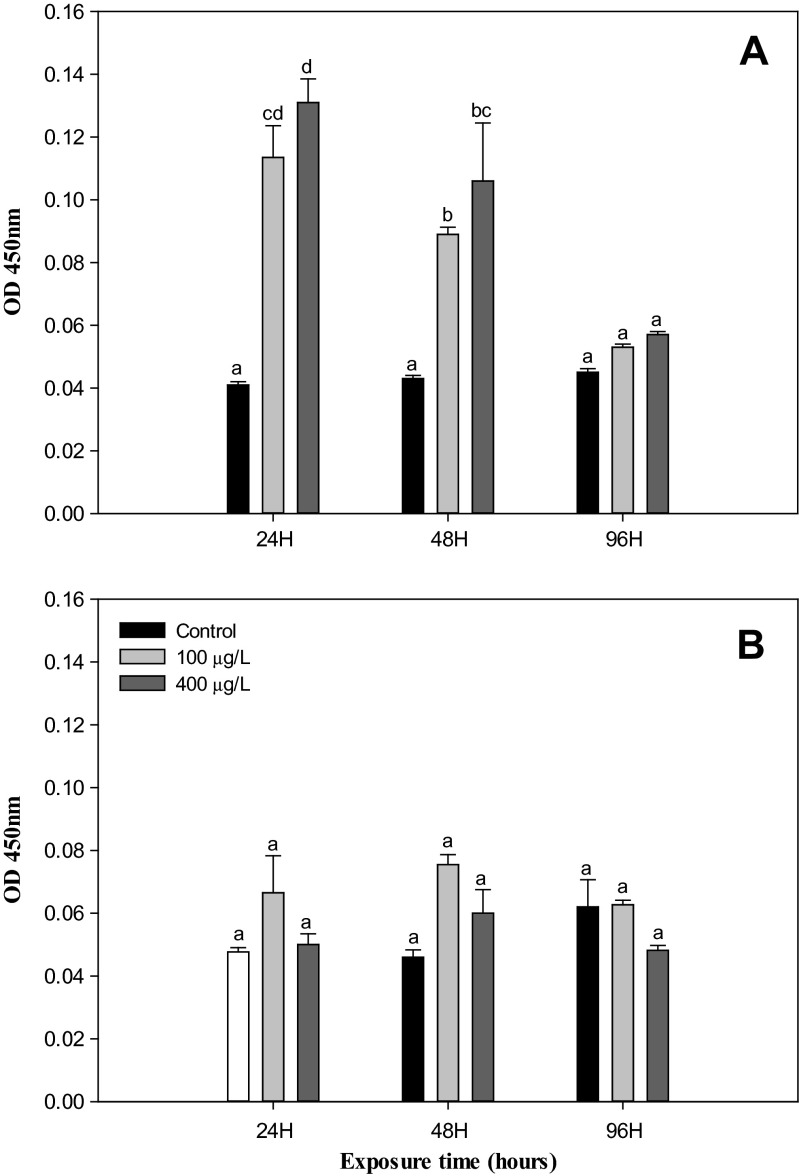
Phagocytosis activity of hemocytes in abalone, *H. discus hannai* Ino, exposed to various NiCl_2_ concentrations at 22 °C (**a**) and 26 °C (**b**) for 96 h. Each point represents a mean value ± SD of three replicates. *Vertical bar* denotes a standard error (*n* = 10). Values with *different superscripts* are significantly different (*P* < 0.05) as determined by Duncan’s multiple range test

In this study, the THC reduced significantly (*P* < 0.05) while the lysozyme, PO, and phagocytosis activity increased significantly (*P* < 0.05) in the hemolymph of *H. discus hannai* exposed to NiCl_2_ (100 and 400 μg/L) when compared to the control at both 22 and 26 °C. However, these immunological parameters were not impacted by the rise in temperature from 22 to 26 °C in nonexposed groups during experimental periods, except for lysozyme activity at 48 h (Figs. 1, 2, 3, and 4).

The invertebrate defense system has been established to depend solely on an innate immune system, in which the circulating hemocytes play key roles, and the THC can reflect the health status of the host (Gopalakrishnan et al. 2009). Previous studies suggested that hemocyte functions can be used as biomarkers to study the effects of pollution (Fisher et al. 2000; Gopalakrishnan et al. 2009). In this study, the THC significantly decreased depending on the NiCl_2_ concentration and water temperature (Fig. 1). Consistent with these results, abalones exposed to benozo(a)pyrene and tributyltin exhibit a decreased THC (Gopalakrishnan et al. 2009, 2011). Ano and Mori (1996) suggested that the decline in the THC is due to inhibition of the mobilization of hemocytes by xenobiotic substances and stressors. Also, as suggested by Vijayavel et al. (2009), Ni might be transported to other organs via the circulating hemolymph. The reduction in the THC might be due to the interference of Ni and thermal stress with hematopoietic tissues, which serve as the production and storage sites for the hemocytes. The hemolymph protein level also has an important role in the maintenance of the stability of hemocytes, oxygen transport, and cell integrity (Vijayavel et al. 2005). However, in this study, the level of hemolymph protein in the abalone exposed to NiCl_2_ during thermal stress did not change significantly compared to the control group (Table 2).

As noted above, the humoral immune parameters, the lysozyme, and PO activity are important humoral defense factors in mollusks. Lysozyme performs the primary role of eliminating pathogens and other invaders in invertebrates and may be involved in the segregation and metabolism of toxic compounds (Lowe and Pipe 1994). In previous studies, a significant inhibition of lysozyme was reported in the abalone *Haliotis diversicolor supertexta* and the clam *Tapes philippinarum* exposed to tributyltin (Zhou et al. 2010; Mattzzo et al. 2002). At 26 °C in this study, the lysozyme activity decreased significantly in the abalone exposed to NiCl_2_ after 96-h exposure. This decrease in the activity of lysozyme indicates an attenuated disease resistance in abalone (Fig. 2). However, at the higher temperature (26 °C), the activity of the lysozyme of abalone hemolymph increased in the control groups after 48-h exposure (Fig. 2). The reason why the lysozyme in group treated with NiCl_2_ was higher than in the controls during the 96 h at 22 °C and the first 48 h at 26 °C possibly results from hormesis (Stjean et al. 2002). As shown in Fig. 3, in the PO activity, a parallel change occurred in both the controls (22 °C) and heated abalone (26 °C) from 24 to 96 h, with a significant increase over time in the abalone exposed to NiCl_2_. Day et al. (2010) reported a similar change in both controls (16 °C) and heated abalone (26 °C) from days 1 to 2, but the PO activity in heat-stressed abalone significantly decreased compared to the controls on day 7. They suggested the long-term suppression of this aspect of the immune function by continued severe heat stress and a possible increase with temperature that was associated with shorter, less severe heat stress. Exposure to benzo(a)pyrene also significantly increased the activity of PO in *H. diversicolor* (Gopalakrishnan et al. 2009). In vertebrates, PO exists in hemolymph as an inactive proenzyme, prophenoloxidase, which can be transformed to the active form, PO, by several microbial polysaccharides, environmental factors, and metal ions (Coles and Pipe 1994; Cárdenas and Dankert 1997). PO can also be released from the circulating hemocytes into hemolymph when the animals are stressed by physical injury or infection (González et al. 2003; Gopalakrishnan et al. 2009). Exposure to xenobiotic substances has been well established to lead to an increase in PO in mollusks (Coles and Pipe 1994; Cheng et al. 2004e; Thiagarajan et al. 2006; Gopalakrishnan et al. 2009), and the results of this study also support earlier reports suggesting that sublethal levels of NiCl_2_ have an impact on the plasma PO of *H. discus hannai*. Nappi et al. (1995) reported that intermediates of PO may generate the superoxide anion. Increased PO due to NiCl_2_ exposure might indirectly produce free radicals that could lead to oxidative stress and cellular damage. Previous studies have shown that thermal stress in marine organisms can significantly alter ROS production and antioxidant enzyme expression (Power and Sheehan 1996; Abele and Puntarulo 2004; Heise et al. 2003; Zoysa et al. 2009).

As observed in the hemolymph PO activity, phagocytosis by hemocytes significantly increased compared to the controls in *H. discus hannai* exposed to Ni during the experimental periods (Figs. 3 and 4). Although an increase in PO activity lasted throughout the experimental period (96 h), the level of phagocytosis recovered to the control level after 96 h at 22 °C but was not significantly different from the start to finish of the experiment at 26 °C (Fig. 4).

Phagocytosis can be affected by environmental parameters in vertebrates (Bayne 1990). In particular, an elevated temperature has been reported to increase phagocytosis and hemocyte activity in *Crassostrea virginica* (Feng and Feng 1974; Foley and Cheng 1975). Day et al. (2010) reported that substantial temporal variation is found in the rate of phagocytosis of abalone hemocytes. On days 1 and 2, the heat-stressed (26 °C) abalone had significantly higher rates, but beyond the first day, no evidence was observed of an effect of heat on the rate of phagocytosis. The authors suggested that this may be an artifact (i.e., an effect of some unforeseen factor, possibly the cessation of feeding on day 1, or lights being turned on without the authors’ knowledge). Dang et al. (2012) reported that THC increased at day 1 and then dropped back to control levels by days 3 and 7 and that antiviral and antibacterial activity tended to be elevated in the higher-temperature (21 and 24 °C)-treated groups compared to the control group (18 °C).

In general, xenobiotic substances suppress the values of most immune parameters in abalone. For example, phagocytosis activity decreased significantly with increasing concentrations of Ni (400–800 μg/L) in the hemocytes of the mud crab *S. serrata* (Vijayavel et al. 2009). However, a significant discrepancy was observed in the immunological parameters of abalone exposed to NiCl_2_ during the thermal stress experienced in this study. This can be explained by the results of the following studies. Previous studies on *Macrobrachium rosenbergii* indicated that PO, phagocytic activity, and the clearance efficiency of bacteria were significantly higher for animals reared at 27 and 30 °C than those reared at 20 and 33 °C (Cheng and Chen 2000; Cheng et al. 2003). Sauve et al. (2002) reported that the effect of heavy metals on immune responses differed due to the period of exposure to the heavy metals. Similarly, copper and Hg inhibited the immune responses in *Perna viridis*, although they recovered from the toxic effect after prolonged exposure to these metals (Thiagarajan et al. 2006). The ability to eliminate bacteria from the circulating hemolymph and the number of hemocytes initially decreased after exposure to aluminum (Al) and a bacterial challenge but then also recovered after long-term exposure to Al (Ward et al. 2006). These studies suggest that “mild stressors” can cause an apparent immune stimulation, but a concurrent increased susceptibility to infectious disease occurs. An important aim of future research should be to identify the impacts on immune system function following exposure to complex stressors (e.g., temperature, heavy metals, and pathogen attacks) that are relevant in assessing on-farm immune functional capacity.

Our results showed that Ni concentrations below 400 μg/L NiCl_2_ were able to stimulate immune responses in abalone. However, complex stressors, such as the thermal variability of breeding water or Ni exposure, can modify an immunological response and can lead to changes in the physiology of host–pollutant interactions in *H. discus hannai*. Ni is often found in the coastal environment, with levels in natural waters ranging from 0.2 to 0.7 μg/L in marine water (Brix et al. 2004). The exact amount of Ni in the marine environment is likely to depend on local factors, and the cause of the mortality-induced suppression of the immune response of abalone may not be due to Ni, even under conditions of climate change.

## References

[CR1] Abele D, Puntarulo S (2004). Formation of reactive species and induction of antioxidant defense systems in polar and temperature marine invertebrates and fish. Comp Biochem Physiol.

[CR2] Alam MK, Maughan OE (1982). The effect of malathion, diazinon, and various concentrations of zinc, copper, nickel, lead, iron, and mercury on fish. Biol Trace Elem Res.

[CR3] Ano H, Mori K (1996). Interaction between hemocytes and plasma is necessary for haemolymph coagulation in the spiny lobster, Panulirus japonicas. Comp Biochem Physiol.

[CR4] Asokan R, Arumugam M, Mullainadhan P (1997). Activation of prophenoloxidase in plasma and hemocytes of the marine mussel *perna viridis Linnaeus*. Develop Comparat Immunol.

[CR5] Atchison GJ, Henry MG, Sandheinrich MB (1987). Effects of metals on fish behavior: a review. Environ Biol Fishes.

[CR6] Auffret M, Mujdzic N, Corporeau C, Moraga D (2002). Xenobiotic induced immunomodulation in the European flat oyster, *Ostrea edulis*. Mar Environ Res.

[CR7] Bayne CJ (1990). Phagocytosis and non-self-recognition in invertebrates phagocytosis appears to be an ancient line of defense. BioSci.

[CR8] Bielmyer GK, DeCarlo C, Morris C, Carrigan T (2013). The influence of salinity on acute nickel toxicity to the two euryhaline fish species, *Fundulus heteroclitus* and *Kryptolebias marmoratus*. Environ Toxicol Chem.

[CR9] Braid BA, Moore JD, Robbins TT, Hedrick RP, Tjeerdema RS, Friedman CS (2005) Health and survival of red abalone, *Haliotis rufescens*, under varying temperature, food supply,and exposure to the agent of withering syndrome. J Invert Pathol 89(3):219–231 10.1016/j.jip.2005.06.004 16039668

[CR10] Brix KV, Keithly J, DeForest DK, Laughlin J (2004). Acute and chronic toxicity of nickel to rainbow trout (*Oncorhychus mykiss*). Environ Toxicol Chem.

[CR11] Brousseau P, Pellerin J, Morin Y, Cyr D, Blakley B, Boermans H, Fournier M (2000). Flow cytometry as a tool to monitor the disturbance of phagocytosis in the clam *Mya arenaria* hemocytes following in vitro exposure to heavy metals. Toxicology.

[CR12] Buhl KJ, Hamilton SJ (1991). Relative sensitivity of early life stages of Arctic grayling, Coho salmon, and rainbow trout to nine inorganics. Ecotoxicol Environ Safe.

[CR13] Cárdenas W, Dankert JR (1997). Phenoloxidase specific activity in the red swamp crayfish, *Procambarus clarkia*. Fish Shellfish Immunol.

[CR14] Cheng W, Chen JC (2000). Effects of pH, temperature and salinity on immune parameters of the freshwater prawn *Macrobrachium rosenbergii*. Fish Shellfish Immunol.

[CR15] Cheng W, Chen SM, Wang FI, Hsu PI, Liu CH, Chen JC (2003). Effects of temperature, pH, salinity and ammonia on the phagocytic activity and clearance efficiency of giant freshwater prawn *Marcrobrachium rosenbergii* to Lactococcus garvieae. Aquaculture.

[CR16] Cheng W, Hsiao IS, Hsu CH, Chen JC (2004). Change in water temperature on the immune response of Taiwan abalone *Haliotis diversicolor supertexta* and its susceptibility to *Vibrio paranhaemolyticus*. Fish Shellfish Immunol.

[CR17] Cheng W, Juang FM, Chen JC (2004). The immune response of Taiwan abalone *Haliotis diversicolor supertexta* and its susceptibility to *Vibrio parahaemolyticus* at different salinity levels. Fish Shellfish Immunol.

[CR18] Cheng W, Li CH, Chen JC (2004). Effect of dissolved oxygen on the immune response of Taiwan abalone *Haliotis diversicolor supertexta* and its susceptibility to *Vibrio parahaemolyticus*. Aquaculture.

[CR19] Cheng W, Hsiao IS, Chen JC (2004). Effect of ammonia on the immune response of Taiwan abalone *Haliotis diversicolor supertexta* and its susceptibility to *Vibrio parahaemolyticus*. Fish Shellfish Immunol.

[CR20] Cheng W, Hsiao IS, Chen JC (2004). Effect of nitrite on the immune response of Taiwan abalone *Haliotis diversicolor supertexta* and its susceptibility to *Vibrio parahaemolyticus*. Dis Aquat Organ.

[CR21] Coles N, Pipe RK (1994). Phenoloxidase activity in the haemolymph and haemocyte of the marine mussel *Mytilus edulis*. Fish Shellfish Immunol.

[CR22] Dang VT, Speck P, Benkendorff K (2012). Influence of elevated temperature on the immune response of abalone, *Haliotis rubra*. Fish Shellfish Immunol.

[CR23] Day R, Hooper C, Benkendorff K, Slocombe R, Handlinger J (2010) Investigation on the immunology of stressed abalone. Final report of FRDC project number: 2004/233. University of Melbourne, Australia, pp 1–89

[CR24] Donker MH, Abdel-Lateif HM, Khalil MA, Bayoumi BM, Van Straalen NM (1998). Temperature physiological time, and zinc toxicity in the isopod *Porcelio scaber*. Environ Toxicol Chem.

[CR25] Eisler R (1998) Nickel hazards to fish, wildlife, and invertebrates: a synoptic review. US Geological Survey, Biological Science Report USGS/BRD/BSR-1998-0001

[CR26] Elder JF, Mattraw HC (1984). Accumulation of trace elements, pesticides, and polychlorinated biphenyls in sediments and the clam Corbicula manilensis of the Apalachicola River, Florida. Arch Environ Contam Toxicol.

[CR27] Ellis AE (1990) Lysozyme assay. In: Stolen JS, Fletcher TC, Anderson DP, Roberson BS, van Musiwinkel WB (eds) Techniques in fish immunology. SOS publications, Fairhaven, pp 1-197

[CR28] Feng SY, Feng JS (1974). The effect of temperature on cellular reactions of Crassostrea virginica to the injection of avian erythrocytes. J Inverterbr Pathol.

[CR29] Finney DJ (1971) Probit analysis. Cambridge University Press, Cambridge.

[CR30] Fisher WS, Oliver LM, Winstead JT, Long ER (2000). A survey of oysters *Crassostrea virginica* from Tampa Bay, Florida: association of internal defense measurements with contaminant burdens. Aquat Toxicol.

[CR31] Foley DA, Cheng TC (1975). A quantitative study of phagocytosis by hemolymph cells of the pelecypods *Crassostrea virginica* and *Mercenaria mercenaria*. J Invertebr Pathol.

[CR32] Gagnaire B, Thomas-Guyon H, Renault T (2004). In vivo effects of cadmium and mercury on Pacific oyster, Crassostrea gigas (Thunberg), haemocytes. Fish Shellfish Immunol.

[CR33] Gagne F, Auclair J, Turcotte P, Fournier M, Gagnon C, Sauve S, Blaise C (2008). Ecotoxicity of CdTe quantum dots to freshwater mussels: impacts on immune system, oxidative stress and genotoxicity. Aquat Toxicol.

[CR34] González AL, Martinez MAN, Albores FV, Valle FA, Mungaray MR (2003). Phenoloxidase activity in larval and juvenile homogenates and adult plasma and haemocytes of bivalve molluscs. Fish Shellfish Immunol.

[CR35] Gopalakrishnan S, Thilagam H, Huang WB, Wang KJ (2009). Immunomodulation in the marine gastropod *Haliotis diversicolor* exposed to benzo(a)pyrene. Chemosphere.

[CR36] Gopalakrishnan S, Huang WB, Wang QW, Wu ML, Liu J, Wang KJ (2011). Effect of tributyltin and benzo(a)pyrene on the immune-associated activities of hemocytes and recovery responses in the gastropod abalone, *Haliotis diversicolor*. Comp Biochem Physiol.

[CR37] Harkin A, Hynes MJ, Masterson E, Kelly JP, O’Donnell JM, Connor TJ (2003). A toxicokinetic study of nickel-induced immunosuppression in rats. Immunopharmacol Immunotoxicol.

[CR38] Harvell D, Altizer S, Cattadori IM, Harrington L, Weil E (2008). Climate change and wildlife diseases: when does the host matter the most?. Ecology.

[CR39] Heise K, Puntarulo S, Portner HO, Abele D (2003). Production of reactive oxygen species by isolated mitochondria of the Antarctic bivalve *Laternula elliptica* (King and Broderip) under heat stress. Comp Biochem Physiol.

[CR40] Hoegh-Guldberg O, Bruno JF (2010). The impact of climate change on the world’s marine ecosystems. Science.

[CR41] Khan MAQ, Ahmed SA, Salazar A, Gurumendi J, Khan A, Vargas M, von Catalin B (2007). Effect of temperature on heavy metal toxicity to earthworm *Lumbricus terrestris* (Annelida: Oligochaeta). Environ Toxicol.

[CR42] Khangarot BS, Ray PK (1990). Acute toxicity and toxic interaction of chromium and nickel to common guppy *Poecilia reticulate* (Peters). Bull Environ Contam Toxicol.

[CR43] Kong X, Wang G, Li S (2012). Effects of low temperature acclimation on antioxidant defense and ATPase activities in the muscle of mud crab (*Scylla paramamosain*). Aquaculture.

[CR44] Lee KK, Liu PC, Chen YC, Haung CY (2001). The implication of ambient temperature with the outbreak of vibriosis in cultured small abalone *Haliotis diversicolor supertexa* Lischke. J Thermal Biol.

[CR45] Lee DC, Park YC, Jeon CY, Yang JY, Hur YB, Kim JW, Cho KC (2013). A report on the 2012 mass summer mortalities of black rockfish, *Sebastes schlegeli* in the Southeast Sea, Korea. J Fish Pathol.

[CR46] Lejeusne C, Chevaldonné P, Pergent-Martini C, Boudouresque CF, Pérez T (2010). Climate change effects on a miniature ocean: the highly diverse, highly impacted Mediterranean Sea. Trends Ecol Evol.

[CR47] Lowe DM, Pipe RK (1994). Contaminant induced lysosomal membrane damage in marine mussel digestive cells: an in vitro study. Aquat Toxicol.

[CR48] Malham SK, Lacoste A, Gelebart F, Cueff A, Poulet SA (2003). Evidence for a direct link between stress and immunity in the mollusk *Haliotis tuberculate*. J Exp Zool.

[CR49] Martello LB, Friedman CS, Tjeerdema RS (2000). Combined effects of pentachlorophenol and salinity stress on phagocytic and chemotactic function in two species of abalone. Aquat Toxicol.

[CR50] Mattzzo V, Ballarin L, Marine MG (2002). In vitro effects of tributyltin on functional responses of haemocytes in the clam *Tapes philippinarum*. Appl Organomet Chem.

[CR51] Muyssen BTA, Brix KV, DeForest DK, Janssen CR (2004) Nickel essentiality and homeostasis in aquatic organisms. Environ Rev 12(2):113–131

[CR52] Nappi AJ, Vass E, Frey F, Carton Y (1995). Superoxide anion generation in Drosophila during melanotic encapsulation of parasites. Eur J Cell Biol.

[CR53] Nguyen VT, Qian ZJ, Ryu B, Kim KN, Kim D, Kim YM, Jeon YJ, Park WS, Choi IW, Kim GH, Je JY, Jung WK (2013). Matrix metalloproteinases (MMPs) inhibitory effects of an octameric oligopeptide isolated from abalone *Haliotis discus hannai*. Food Chem.

[CR54] Niyogi S, Brix KV, Grosell M (2014). Effects of chronic waterborne nickel exposure on growth, ion homeostasis, acid–base balance, and nickel uptake in the freshwater pulmonate snail, *Lymnaea stagnalis*. Aquat Toxicol.

[CR55] Ortuno J, Esteban MA, Meseguer J (2002). Lack of effect of combining different stressors on innate immune response of seabream. Vet Immunol Immunopathol.

[CR56] Othman MS, Amalina RN, Nadzifah Y (2012). Toxicity of metals to a freshwater snail, *Melanoides tuberculata*. Sci World J.

[CR57] Pane E, Richards J, Wood C (2003). Acute waterborne nickel toxicity in the rainbow trout (*Oncorhynchus mykiss*) occurs by a respiratory rather than ionregulatory mechanism. Aquat Toxicol.

[CR58] Pane E, Haque A, Wood C (2004). Mechanistic analysis of acute, Ni-induced respiratory toxicity in the rainbow trout (*Oncorhynchus mykiss*): an exclusively branchial phenomenon. Aquat Toxicol.

[CR59] Pipe PK, Coles JA, Carissan FMM, Ramanathan K (1999). Copper induced immunomodulation in the marine mussel, *Mytilus edulis*. Aquat Toxicol.

[CR60] Power A, Sheehan D (1996). Seasonal variation in the antioxidant defense of gill and digestive gland of the blue mussel, Mytilis edulis. Comp Biochem Physiol.

[CR61] Prophete C, Carlson EA, Duffy YL, Steinetz B, Lasano S, Zelikoff JT (2006). Effects of elevated temperature and nickel pollution on the immune status of *Japanase medaka*. Fish Shellfish Immunol.

[CR62] Rand GM, Petrocelli SR (1985) In: Rand GM, Petrocelli SR (eds) Fundamentals of aquatic toxicology: methods and applications. Hemisphere Publishing Corp., Washington, pp 1–31

[CR63] Sauve S, Brousseau P, Pellerin J, Morin Y, Senecal L, Goudreau P (2002). Phagocytic activity of marine and freshwater bivalves: in vitro exposure of hemocytes to metals (Ag, Cd, Hg and Zn). Aquat Toxicol.

[CR64] Saxena OP, Parashari A (1983). Comparative study of the toxicity of six heavy metals to *Channa punctatus*. J Environ Biol.

[CR65] Sjursen H, Holmstrup M (2004). Cold and drought stress in combination with pyrene exposure: studies with *Protaphorura armata* (Collembola: Onychiuridae). Ecotoxicol Environ Saf.

[CR66] Stjean SD, Pelletier E, Courtenay SC (2002). Very low levels of waterborne butyltins modulate hemocyte function in the blue mussel *Mytilis edulis*. Mar Ecol Prog Ser.

[CR67] Sun HX, Dang Z, Xia Q, Tang WC, Zhang GR (2011). The effect of dietary nickel on the immune responses of *Spodoptera litura* Fabricius larvae. J Insect Physiol.

[CR68] The Annual Book of ASTM standards (1980) Standard practice for conducting acute toxicity tests with fishes, macroinvertebrates and amphibians. Designation E729-80. American Society for Testing and Materials, Philadelphia, PA, USA pp. 25

[CR69] Thiagarajan R, Gopalakrishnan S, Thilagam H (2006). Immunomodulation in the marine green mussel *Perna viridis* exposed to sub-lethal concentrations of Cu and Hg. Arch Environ Contam Toxicol.

[CR70] Vijayavel K, Anbuselvam C, Balasubramanian MP (2005). Naphthalene-induced hematological disturbances and oxidative stress in an estuarine edible crab, *Scylla serrata*. Environ Toxicol.

[CR71] Vijayavel K, Gopalakrishnan S, Thiagarajan R, Thilagam H (2009). Immunotoxic effects of nickel in the mud crab *Scylla serrata*. Fish Shellfish Immunol.

[CR72] Virk S, Sharma RC (1995). Effect of nickel and chromium on various life stages of Cyprinus carpio Linn. Indian J Ecol.

[CR73] Wanger E, Bosakowski T, Intelmann S (1997). Combined effects of temperature and high pH on mortality and the stress response of rainbow trout after stocking. Trans Am Fish Soc.

[CR74] Ward RJS, McCrohan CR, White KN (2006). Influence of aqueous aluminum on the immune system of the freshwater crayfish, *Pacifastacus leniusculus*. Aquat Toxicol.

[CR75] Yue F, Pan L, Xie P, Zheng D, Li J (2010). Immune responses and expression of immune-related genes in swimming carb *Portunus trituberculatus* exposed to elevated ambient ammonia-N stress. Comp Biochem Physiol.

[CR76] Zelikoff JT, Dean J, Luster M, Munson A, Kimber I (1994). Fish immunotoxicology. Immunotoxicology and immunopharmacology.

[CR77] Zelikoff JT, Wang W, Islam N, Twerdok LE, Curry M, Beaman J, Ostrander G (1996). Assays of reactive oxygen intermediates and antioxidant enzymes: potential biomarkers for predicting the effects of environmental pollution. Techniques in aquatic toxicology.

[CR78] Zhang D, Shen J, Wang C, Zhang X, Chen J (2008). GSH-dependent iNOS and HO-1 mediated apoptosis of human Jurkat cells induced by nickel(II). Environ Toxicol.

[CR79] Zhou J, Cai ZH, Zhu XS, Li L, Gao YF (2010). Innate immune parameters and haemolymph protein expression profile to evaluate the immunotoxicity of tributyltin on abalone (*Haliotis diversicolor supertexa*). Develop Comp Immunol.

[CR80] Zoysa MD, Whang I, Lee YD, Lee SK, Lee JS, Lee JH (2009). Transcriptional analysis of antioxidant and immune defense genes in disk abalone (*Haliotis discus discus*) during thermal, low-salinity and hypoxic stress. Comp Biochem Physiol.

